# Clinical characteristics predicting internal neurofibromas in 357 children with neurofibromatosis-1: results from a cross-selectional study

**DOI:** 10.1186/1750-1172-7-62

**Published:** 2012-09-03

**Authors:** Emilie Sbidian, Smaïl Hadj-Rabia, Vincent M Riccardi, Laurence L Valeyrie-Allanore, Sébastien Barbarot, Olivier Chosidow, Salah Ferkal, Diana Rodriguez, Pierre Wolkenstein, Sylvie Bastuji-Garin

**Affiliations:** 1 Université Paris Est (UPEC), LIC EA4393 (Laboratoire d’Investigation Clinique), F-94010, Créteil, France; 2 Assistance Publique-Hôpital Paris (AP-HP), Hôpital Henri-Mondor, Service de Dermatologie, Créteil, F-94010, France; 3 Assistance Publique-Hôpital Paris (AP-HP), Hôpital Henri-Mondor, Pôle Recherche Clinique-Santé Publique, Créteil, F-94010, France; 4 Assistance Publique-Hôpital Paris (AP-HP), Hôpital Henri-Mondor, Centre de référence des Neurofibromatoses, Créteil, F-94010, France; 5 Assistance Publique-Hôpital Paris (AP-HP), Hôpital Necker-Enfants malades, Centre de Référence des Maladies Génétiques à Expression Cutanée (MAGEC) et Service de Dermatologie, Université Paris V Descartes, Paris, F-75015, France; 6 The Neurofibromatosis Institute, La Crescenta, CA, USA; 7 Service de Dermatologie, CHU Hôtel Dieu, Nantes, F-44200, France; 8 Université Paris Est (UPEC), F-94010, Créteil, France; 9 INSERM, Centre d’Investigation Clinique 006, Créteil, F-94010, France; 10 Assistance Publique-Hôpital Paris (AP-HP), Hôpital Trousseau, Service de Neuropédiatrie, Université Pierre et Marie Curie, Paris, F-75571, France; 11 Assistance Publique-Hôpital Paris (AP-HP), Hôpital Henri-Mondor, Unité de Recherche Clinique (URC), Créteil, F-94010, France; 12 Service de Santé Publique, Hôpital Henri-Mondor, 51 avenue du Maréchal de Lattre de Tassigny, Creteil Cedex, 94010, France

**Keywords:** Neurofibromatosis 1, Internal neurofibromas, Subcutaneous neurofibromas, Children, Cross-sectional study

## Abstract

**Objective:**

To identify clinical characteristics associated with internal neurofibromas in children with NF1, as a means of ensuring the early identification of patients at high risk for malignant peripheral nerve-sheath tumors developed from preexisting internal neurofibromas.

**Patients and methods:**

We used data from two NF1 populations, in France and North America, respectively. The French database comprised 1083 patients meeting NIH diagnostic criteria for NF1 and the Neurofibromatosis Institute Database of North America comprised 703 patients. Patients younger than 17 years of age were eligible for our study if they had been evaluated for internal neurofibromas using computed tomography and/or magnetic resonance imaging. Clinical characteristics associated with internal neurofibromas by univariate analysis (*P* ≤ 0.15) were entered into a multiple logistic regression model after checking for potential interactions and confounding. Multiple imputation was used for missing values.

**Results:**

Among the 746 children in the two databases, 357 (48%) met our inclusion criteria. Their mean age was 7.7 ± 5.0 years and there were 192 (53.8%) males. Internal neurofibromas were present in 35 (9.8%) patients. Internal neurofibromas developed earlier in females than in males and their prevalence increased during adolescence. Factors independently associated with internal neurofibromas were age (OR = 1.16 [1.07-1.27]), xanthogranulomas (OR = 5.85 [2.18-15.89]) and presence of both subcutaneous and plexiform neurofibromas (OR = 6.80 [1.52-30.44]).

**Conclusions:**

Several easily recognizable clinical characteristics indicate a high risk of internal neurofibromas in children with NF1 and, therefore, a need for very close monitoring.

## Background

Neurofibromatosis-1 (NF1 [MIM 162200]) is a common autosomal dominant disorder with an incidence of 1 in 2500–3000 births and a prevalence of 1 in 4000 [1]. NF1 is associated with increased morbidity and mortality rates [2,3]. In spite of tremendous progress in understanding and treating NF1, there continues to be inconsistency, if not confusion about the various types of neurofibromas. Our interest in this article is to sensitize clinicians to NF1 neurofibromas that are not obvious by inspection and/or palpation of the skin. That is, the focus is on internal neurofibromas. An internal neurofibroma simply is a neurofibroma that is not appreciated by physical examination. Part of the problem is that some neurofibromas that may have internal components may also be apparent externally. In particular, both the large *diffuse plexiform neurofibroma* and the large *nodular plexiform neurofibroma*, as described by Riccardi [4], corresponding to Masson’s *diffuse neurofibroma* and *encapsulated neurofibroma*, respectively, as utilized by Tucker and coworkers [5,6], can have both external and internal components. In the present article we are reserving the term, internal neurofibroma, for those neurofibromas not having an apparent external component. By definition, both cutaneous neurofibromas and subcutaneous neurofibromas are excluded from being internal neurofibromas. Most specifically, the term, plexiform neurofibroma, does not on its own afford distinction from or inclusion in what we are specifying here as an internal neurofibroma.

Internal neurofibromas are among the main causes of life-threatening events in patients with NF1 [4,7]. Ten percent of internal neurofibromas undergo transformation to malignant peripheral nerve-sheath tumors (MPNSTs) [8], a leading cause of death in adults with NF1 [2,3]. Clinical indicators of MPNST are persistent or increasing pain, enlargement of the tumor, and neurological deficiencies [9]. The diagnosis is often delayed, especially for deep diffuse plexiform or internal neurofibromas as imaging studies are not performed routinely as part of the follow-up of patients with NF1 [10] but instead are ordered only as clinically indicated [11]. The mean age at diagnosis of MPNST is younger in patients with NF1 than in unaffected individuals [8,12-14]. MPNSTs may develop before 30 years of age [8,12] and even in childhood [13]. Moreover internal neurofibromas grow faster in young patients [15]. In previous work, we developed a simple scoring system (the NF1Score) that accurately predicted the presence of internal neurofibromas at risk for transformation to MPNST in adults with NF1 [16]. Four characteristics were independently associated with internal neurofibromas: presence of subcutaneous neurofibromas, fewer than 6 café-au-lait spots, absence of cutaneous neurofibromas, and age ≤30 years. However, the NF1Score was developed in patients aged 17 years or older. Consequently, a specific study was needed in pediatric patients. The clinical expression of NF1 varies across the life span in a given patient [11,17]. For instance, café-au-lait spots are present within the first year of life and cutaneous or subcutaneous neurofibromas are usually present by adolescence [1,17,18].

The purpose of this study was to identify clinical characteristics associated with internal neurofibromas in children with NF1. Such clinical characteristics could serve to ensure the early identification of children with NF1 who require particularly close monitoring for internal neurofibromas.

## Patients and methods

### Population and study samples

**Figure 1:**
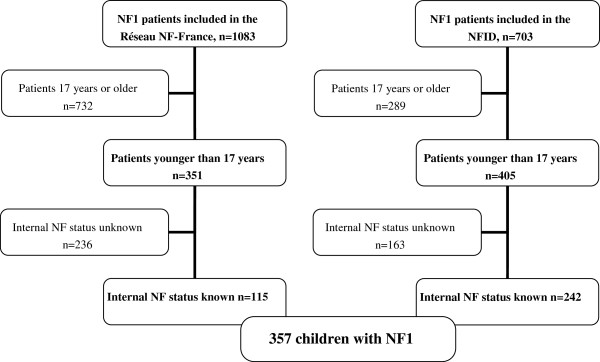
Flow chart.

The study was performed using data from two NF1 populations, one in France and the other in North America. Patients meeting the diagnostic criteria for NF1 established at the National Institutes of Health Consensus Development Conference [19] were included prospectively by the NF-France network (Réseau NF-France) from 2002 to 2005 [20] and the Neurofibromatosis Institute Database (NFID) from 1977 to 1996 [4]. The NF-France database was a French Clinical Research Program entitled “Study of expressivity of neurofibromatosis-1: constitution of a phenotype-genotype database” [20]. The NFID is a collaborative system for collecting demographic information, clinical signs and symptoms, basic measurements, and psychosocial assessments of individuals and families with neurofibromatosis [4]. The NF-France database included 1083 patients and the NFID 703 patients. For the present study, patients were eligible if they were younger than 17 years of age and had undergone imaging studies to look for internal neurofibromas because of symptoms such as pain or neurologic deficits or when there was evidence of internal neurofibromas on other imaging studies (Figure 1). In all, 357 children met these criteria.

The study was approved by the Île-de-France IX Ethics committee Paris, France. Informed consent was obtained from all patients. The study adhered to Declaration of Helsinki guidelines.

### Data collection

The demographic information (age, sex, and whether the NF1 was familial or sporadic) and clinical characteristics recorded in the databases were collected during routine clinical assessments at neurofibromatosis clinics (Table 1). Among the clinical characteristics, we selected those known to be associated with mortality in children with NF1 (plexiform neurofibromas) or showing trends toward an association with mortality in NF1 patients (subcutaneous neurofibromas, absence of cutaneous neurofibromas, and short stature) [21]. Special attention was also given to clinical characteristics independently associated with internal neurofibromas in adult NF1 patients, namely, fewer than 6 café-au-lait spots, age ≤30 years (a criterion met by all our patients), and subcutaneous and cutaneous neurofibromas [16]. Most of the other clinical characteristics selected for our study were easily identified by physical examination, with the exception of Lisch nodules, which were evaluated by slit lamp examination. Detailed information was available on the dermatological characteristics, namely, freckles, plexiform neurofibromas, and xanthogranulomas (previous or current diagnosis). We recorded the following characteristics as present or absent: facial asymmetry, orthopedic complications (nonunion, dysplasia), hypertension, and macrocephaly.

**Table 1:** General characteristics of the 357 children with neurofibromatosis-1 included in the study

**Clinical characteristics (n = missing data)**	**n (%)**
Male gender	192 (53.8)
Age at the first visit in years, mean ± SD (range)	7.7 ± 5.0 (0.01-16.9)
Familial case	183 (51.3)
**Internal neurofibromas**	**35 (9.8)**
Subcutaneous neurofibromas (n = 17)	72 (20.2)
Cutaneous neurofibromas	153 (42.9)
Plexiform neurofibromas (n = 184)	83 (23.3)
Café-au-lait spots	354 (99.2)
Freckles (n = 11)	219 (61.3)
Lisch nodules (n = 84)	160 (44.8)
Xanthogranulomas (n = 68)	14 (3.9)
Short stature (n = 32)	36 (10.1)
Macrocephaly (n = 21)	85 (23.8)
Nonunion	13 (3.6)
Dysplasia (n = 8)	31 (8.7)
Facial asymmetry (n = 72)	23 (6.4)
Hypertension (n = 28)	3 (0.8)

The data are number (%) unless otherwise specified.

### Study definitions

Cutaneous neurofibromas are exophytic tumors that move with the skin on examination [4]. Subcutaneous neurofibromas lie deeper in the skin, do not move with the skin, and are firm and sometimes tender to palpation [4]. A plexiform neurofibroma is an area of thick hypertrophic skin with tissue hyperpigmentation overlying a subcutaneous tumor [4]. Xanthogranulomas are soft, flat, yellow-to-pink papules. Macrocephaly was defined as a head circumference 2 SDs or more above the age- and sex-matched population mean. Short stature was defined as a height 2 SDs or more below the age- and sex-matched population mean.

### Classification of patients: identification of internal neurofibromas

All the study patients had been evaluated for internal neurofibromas using computed tomography (CT) and/or magnetic resonance imaging (MRI). The presence of internal neurofibromas was coded 1 in the databases.

### Statistical analysis

Quantitative variables are described as mean ± standard deviation (SD). Age was not converted to a categorical variable. Qualitative variables are described as number (%). All tests were two-tailed and *P* values <0.05 were considered statistically significant.

The characteristics of patients with and without internal neurofibromas were compared in univariate analyses. Odds ratios (ORs) were estimated with their 95% confidence intervals (95% CIs), using logistic regression models. ORs were adjusted for age because the prevalence of several NF1 characteristics varies with age (number of café-au-lait spots and cutaneous and subcutaneous neurofibromas). Potential interactions were assessed by pairwise analyses and confounding by fitting multiplicative models. Variables yielding *P* values smaller than 0.15 in the univariate analyses were entered into a multiple logistic regression model. The final model included the variables independently associated with the presence of internal neurofibromas.

We first conducted an analysis without the individuals who had missing data (complete-case analysis). We then estimated the missing values for the co-variates independently associated with the internal neurofibromas in the final model, using the multiple-multivariate-imputations-by-chained-equations procedure in STATA [22], with the missing-at-random assumption. We used all predictors together to impute the missing data values, and we independently analyzed 10 copies of the data using 10 cycles of regression. Logistic regression for binary variables and multinomial logistic regression for categorical variables with k > 2 classes were used to impute missing values.

We conducted all statistical analysis using STATA Statistical Software (version 11.0, StataCorp LP, College Station, TX, USA) and LogXact-8 software (2007, CYTEL Inc. Cambridge, MA, USA).

## Results

### Study population

**Figure 2:**
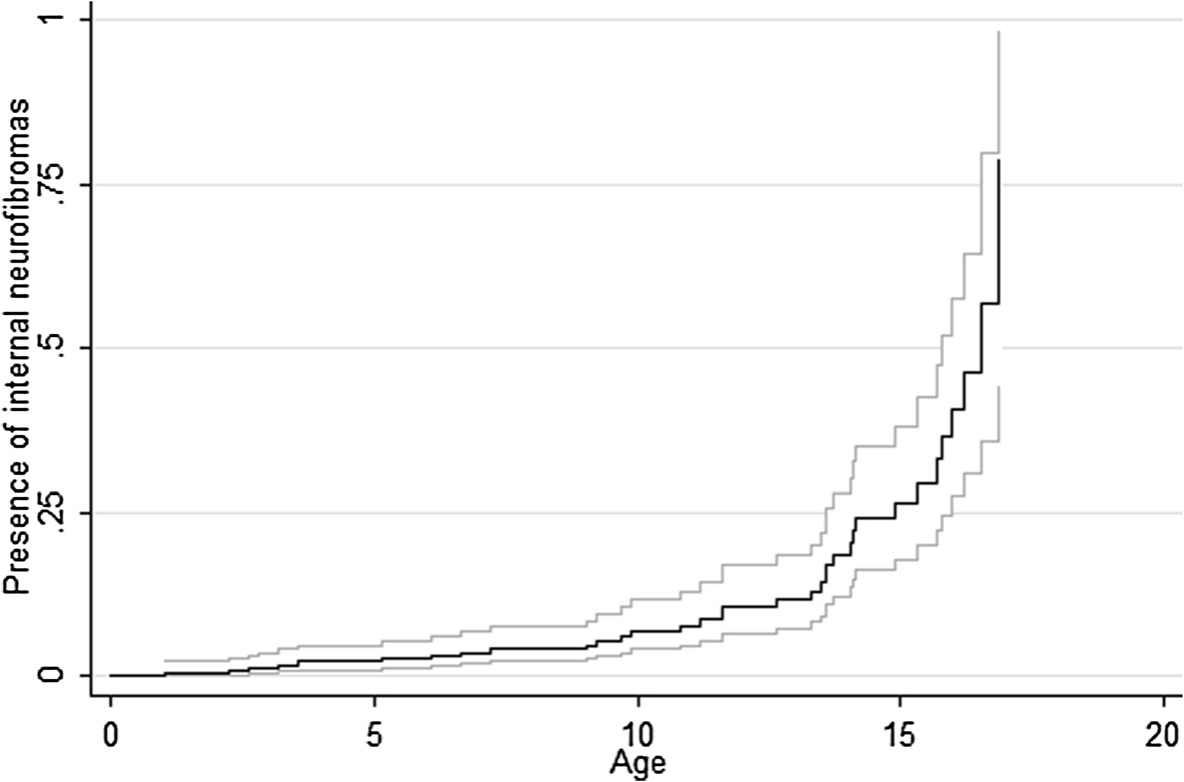
Probability of having internal neurofibromas with the 95% Confidence Interval.

**Figure 3:**
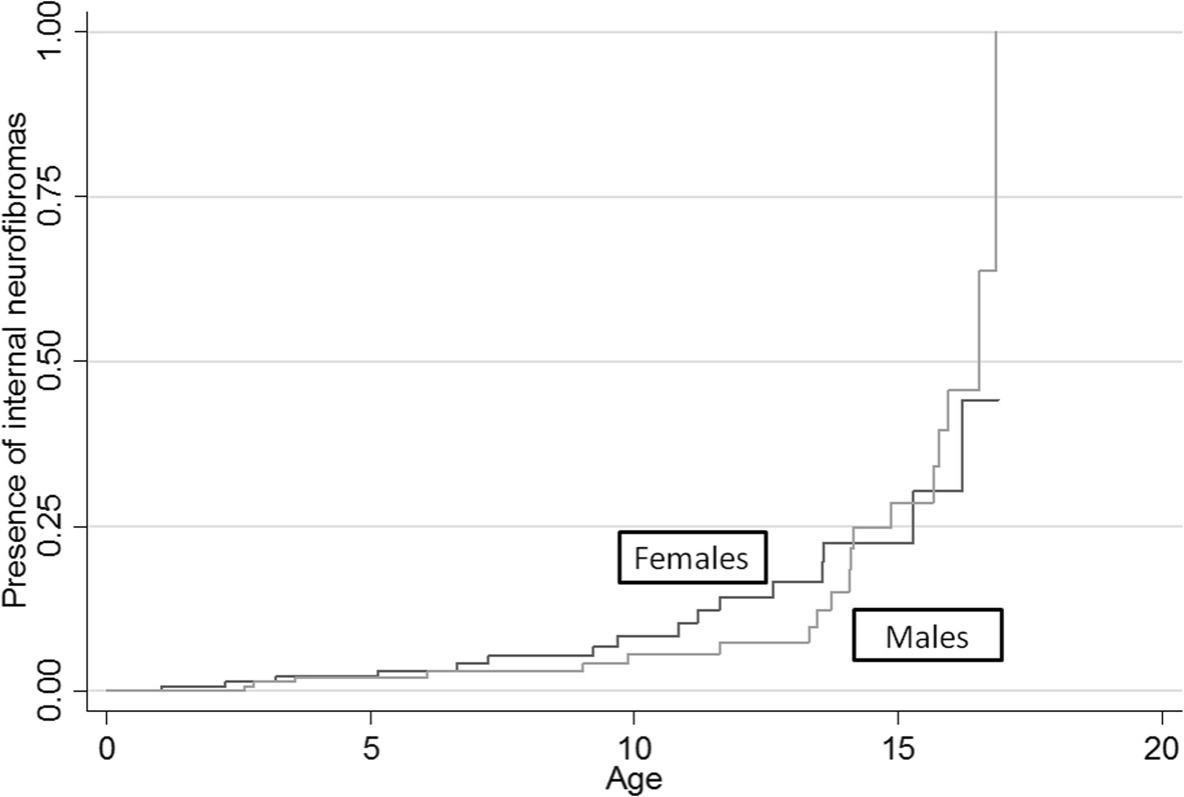
Probability of having internal neurofibromas stratified by sex (females black line and males grey line).

Table 1 reports the main characteristics of the 357 children with NF1 included in our study. Mean age was 7.7 (±5.0) years (range, 0.01-16.9), and there were 192 (53.8%) males. Internal neurofibromas were present in 35 (9.8%) patients. The prevalence of internal neurofibromas increased during adolescence (Figure 2). Internal neurofibromas developed earlier in females than in males (Figure 3).

### Characteristics associated with internal neurofibromas

Table 2 compares the clinical characteristics in the patients with and without internal neurofibromas. By univariate analysis, five variables were associated or nearly associated with internal neurofibromas: age (continuous variable), subcutaneous neurofibromas, plexiform neurofibromas, Lisch nodules, and xanthogranulomas.

**Table 2:** Univariate analysis of selected characteristics for associations with the presence of internal neurofibromas in 357 children with neurofibromatosis-1

	**Internal neurofibromas**		**Odds ratio (95% CI)†**	***P *value‡**
	**No**	**Yes**		
	**n = 322**	**n = 35**		
Male gender	148 (46)	16 (46)	1.01 (0.49-2.07)	0.977
**Age (years)**	7.35 ± 4.87	10.67 ± 4.81	**1.14**^**a**^**(1.07-1.23)**	**<10**^**-4**^
Familial cases	166 (51)	17 (49)	0.61 (0.29-1.27)	0.184
**Subcutaneous neurofibromas (n = 305/35)**	53 (17)	19 (54)	**4.51 (2.13-9.55)**	**<10**^**-4**^
Cutaneous neurofibromas	139 (43)	14 (40)	0.78 (0.37-1.64)	0.512
**Plexiform neurofibromas (n = 140/33)**	**60 (43)**	**19 (70)**	**3.60 (1.55-8.34)**	**0.003**
Café-au-lait spots	319 (99)	35 (100)	0.81 (0.04 - ∞)	1.000
Freckles (n = 312/34)	194 (56)	13 (28)	1.09 (0.47-2.52)	0.840
**Lisch nodules (n = 250/23)**	**151 (60)**	**9 (39)**	**0.35 (0.14–0.86)**	**0.05**
**Xanthogranulomas (n = 257/32)**	**10 (4)**	**4 (13)**	**6.60 (1.69-25.74)**	**0.007**
Short stature (n = 293/32)	32 (11)	4 (13)	1.00 (0.32-3.13)	0.996
Macrocephaly (n = 302/34)	12 (35)	73 (24)	1.65 (0.76-3.54)	0.203
Nonunion	1 (3)	12 (4)	1.8 (0.3-10.2)	0.684
Dysplasia (=314/35)	27 (9)	4 (11)	1.06 (0.34-3.35)	0.914
Facial asymmetry (n = 261/24)	20 (8)	3 (12)	1.23 (0.45-6.93)	0.419
Hypertension (n = 298/31)	2 (1)	1 (3)	5.41 (0.40-73.93)	0.205

**†**Odds ratio with 95% confidence interval (95% CI) by logistic regression adjusted for age.

^a^OR with 95% CI giving the risk increase for a 1-year increase in age.

**‡***P* value by logistic regression, Data are numbers (%) or means ± 1 standard deviation.

The presence of Lisch nodules was strongly associated with the other variables and was not independently associated with internal neurofibromas in the multivariate analysis. The study of potential interactions between variables showed a effect modification between plexiform neurofibromas and subcutaneous neurofibromas (*P* = 0.10). To facilitate the interpretation of the model, the usual assessment of interaction effects is done by entering an additional variable composed of the product of the two variables [23]. So, we replaced the interaction term by a composite variable: no subcutaneous and no plexiform neurofibromas (reference category), either subcutaneous or plexiform neurofibromas, and both subcutaneous and plexiform neurofibromas. We chose these three categories because subcutaneous and plexiform neurofibromas had closely similar OR values by univariate analysis (Table 2).

By multivariate analysis, three characteristics were independently associated with internal neurofibromas: age, presence of xanthogranulomas and presence of both subcutaneous and plexiform neurofibromas (Table 3).

**Table 3:** Characteristics independently associated with internal neurofibromas in the multivariate analysis on complete cases (n = 184) and after multiple imputation (n = 357)

	**Complete-case analysis**^**a**^**, n = 184**			**Imputed data**^**b**^**, n = 357**		
	**Odds ratios**	**95% CI†**	***P *value‡**	**Odds ratios**	95% CI*	*P *value**
**Age**	**1.12**^**x**^	1.02-1.23	0.012	**1.16**^**x**^	1.07-1.27	0.001
**Subcutaneous/plexiform NFs**						
**- None**	**1**			**1**		
**- Either subcutaneous or plexiform NFs**	**1.15**	0.42-3.15	0.781	**1.06**	0.40-2.80	0.901
**- Both subcutaneous and plexiform NFs**	**4.95**	1.80-13.64	0.002	**5.89**	2.18-15.89	<10^-4^
**Xanthogranuloma**	**4.51**	0.94-21.73	0.06	**6.80**	1.52-30.44	0.012

^a^Excluding individuals with missing values.

^b^Missing data imputed using imputation with chain equations.

**†**Odds ratio with 95% confidence interval (95% CI) by logistic regression.

**‡***P* value by logistic regression.

^x^OR with 95% CI giving the risk increase for a 1-year increase in age.

*Odds ratio with 95% confidence interval (95% CI) by logistic regression with multiple imputation.

**P value by logistic regression with multiple imputation.

*NF* neurofibroma.

The general pattern of the results after multiple imputation was similar to that obtained in the patient subset with complete data (Table 3). *P* values were smaller for all three variables independently associated with internal neurofibromas. The OR for the presence of subcutaneous or plexiform neurofibromas was similar between the complete-case and imputed models. The bound categories were thinner as the multivariate analysis included all cases after multiple imputation.

## Discussion

In this study, we identified easily recognizable clinical characteristics associated with internal neurofibromas in children with NF1. By multivariate analysis, age, xanthogranulomas, and presence of both subcutaneous and plexiform neurofibromas were independently associated with internal neurofibromas.

NF1 has been reported to be associated with a 15-year decrease in life expectancy [2]. We recently reported overall excess mortality in a cohort of 1895 patients with NF1 compared to the general population in France [3]. Excess mortality occurred among NF1 patients aged 10 to 20 years (Standard Mortality Ratio, SMR, 5.2; 95% CI, 2.6-9.3; *P* < 10^-4^) and 20 to 40 years (SMR, 4.1; 95% CI, 2.8-5.8; *P* < 10^-4^). MPNSTs were the main cause of death (60%) [3]. Thus, the main concern in the long-term clinical management of patients with NF-1 is the identification of patients at high risk for MPNSTs developed from preexisting internal neurofibromas [2]. Although no effective treatment is available for inoperable MPNSTs, several novel targeted treatments may hold promise [20]. We therefore developed a clinical score, the NF1Score, for predicting the presence of internal neurofibromas in adults with NF1 [16].

Internal neurofibromas grow faster in young children than in adults [15]. Volumetric MRI has been used to assess the growth rate of internal neurofibromas in 49 NF1 patients (median age, 8.3 years; range, 3.3-25) with a median follow up of 34 months. The growth rate per year of internal neurofibromas was significantly greater in the younger patients (<8.3 years) than in the older patients (21.1% versus 8.4%, *P* = 0.001). In addition, if effective treatments are found, they will be more likely to prevent the growth of internal neurofibromas than to reduce their size [24]. Therefore, the benefits will probably be greatest if the treatments are given at the time of most rapid internal neurofibroma growth, that is, in childhood or adolescence. Therefore, the risk factors identified in our study will help to improve the clinical management of children with NF1.

Many factors support the internal validity of our study. First, all patients had a definitive diagnosis of NF1. Selection bias seems unlikely, as the prevalence of NF1 characteristics were consistent with those reported previously in other populations of NF1 patients [25,26]. Our study provided a good external validation as it was performed using two NF1 populations, from France and North America, respectively. However, our patients were recruited at hospitals and may therefore have had greater disease severity compared to the overall NF1 population. An important strength of our study is the accuracy of the information on internal neurofibroma status obtained by using MRI or CT. However, the absence of routine MRI or CT in the study population may constitute a limitation of our study. MRI or CT was performed only when routine imaging studies (chest radiograph and abdominal sonogram) or clinical symptoms suggested the presence of internal neurofibromas. Half our patients did not undergo MRI or CT. This may have resulted in verification bias. Finally, to validate the final model, we used multiple imputation analysis to deal with the missing values. Multiple imputations allow individuals with incomplete data to be included in analyses and improve the validity of the results. The empirical rule of entering only one variable per 10 events in a multivariate analysis model was followed [27].

Internal neurofibromas were present in 35 (9.8%) patients, in keeping with previous data. In a study of 53 children with NF1 who underwent MRI of the entire spine, 7 (13.2%) patients had internal spinal neurofibromas [28]. In our study, older age was independently associated with internal neurofibromas. Excess risk of developing internal neurofibromas seems to occur between the adolescence and the age of to 30 in NF1 patients. Moreover internal neurofibromas increased during adolescence and developed earlier in females, in agreement with previous data [29]. Changes in steroid hormone production may affect the NF1 phenotype. Immunostaining studies have provided support for this hypothesis by identifying the progesterone receptor in neurofibromas [30]. Estrogen and progesterone increased the growth rate of MPNSTs xenografts in mice [31]. Plexiform and subcutaneous neurofibromas were also independently associated with internal neurofibromas in our study, in accordance with earlier work in both adults and children [7,32]. In the MRI study of the spine in children with NF1, characteristics found more often in patients with than without internal neurofibromas included scoliosis (71.4% vs. 30.4%), subcutaneous neurofibromas (71.4% vs. 39.1%), and plexiform neurofibromas (28.6% vs. 8.7%); none of these differences was statistically significant, (*P* = 0.08, *P* = 0.22, and *P* = 0.17, respectively), possibly because statistical power was limited [28]. Furthermore, patients with subcutaneous neurofibromas were at higher risk for mortality in two different NF1 populations, from France [33] and North America [21], respectively. Finally, xanthogranulomas were independently associated with internal neurofibromas in our study. NF1 children with xanthogranulomas are at increased risk for juvenile chronic myelogenous leukemia [34]. Although it should be noted that this study included a small number of patients and has not been confirmed since. To our knowledge, xanthogranulomas have not been previously reported to be associated with internal neurofibromas.

In sum, we identified easily recognizable clinical characteristics that were associated with internal neurofibromas in children with NF1. These risk factors could be used to ensure the early identification of NF1 patients who require particularly close monitoring for internal neurofibromas. In the future, this approach may allow the initiation of treatments at the time of fastest growth of internal neurofibromas, when they are most likely to be effective.

## Abbreviations

aOR: Adjusted odds ratio; CI: Confidence interval; CT: Computed tomography; MPNST: Malignant peripheral nerve sheath tumor; MRI: Magnetic resonance imaging; NF-1: Neurofibromatosis 1; SC-NF(s): Subcutaneous neurofibroma(s); SD: Standard deviation.

## Competing interests

SBG, PW, DR, SF, OC, SB, LVA, VMR, SHR, ES have non-financial interests that may be relevant to the submitted work.

## Authors’ contributions

SBG and PW made substantial contributions to the study conception and design. SBG, PW, SF, SHR, VMR, LVA, ES, DR, and SB made substantial contributions to data acquisition or analysis and to interpretation of the data. ES performed the data analysis under the supervision of SBG. SBG, PW, and ES were involved in drafting the manuscript or revising it critically for important intellectual content. All authors gave final approval of the version submitted for publication.

## Study funding

Grant from the programme hospitalier de recherche clinique (PHRC, AOM 02 108/P 020906), French Ministry of Health.
